# Mutation Spectrum of the *ABCA4* Gene in a Greek Cohort with Stargardt Disease: Identification of Novel Mutations and Evidence of Three Prevalent Mutated Alleles

**DOI:** 10.1155/2018/5706142

**Published:** 2018-04-30

**Authors:** Kamakari Smaragda, Kokkinou Vassiliki, Koutsodontis George, Stamatiou Polixeni, Giatzakis Christoforos, Anastasakis Anastasios, Aslanides Ioannis Minas, Koukoula Stavrenia, Panagiotoglou Theoni, Datseris Ioannis, Tsilimbaris K. Miltiadis

**Affiliations:** ^1^Ophthalmic Genetics Unit, OMMA Ophthalmological Institute of Athens, Athens, Greece; ^2^DNAbiolab, Cretan Center for Research and Development of Applications on Genetics and Molecular Biology, Heraklion, Crete, Greece; ^3^Department of Clinical Electrophysiology of Vision, Athens Eye Hospital, Glyfada, Athens, Greece; ^4^Department of Ophthalmology, Emmetropia Mediterranean Eye Institute, Heraklion, Crete, Greece; ^5^Ophthalmica Institute of Ophthalmology and Microsurgery, Thessaloniki, Greece; ^6^Department of Ophthalmology, University of Crete School of Medicine, Heraklion, Greece; ^7^Department of Retinal Disorders, OMMA Ophthalmological Institute of Athens, Athens, Greece

## Abstract

**Aim:**

To evaluate the frequency and pattern of disease-associated mutations of *ABCA4* gene among Greek patients with presumed Stargardt disease (STGD1).

**Materials and Methods:**

A total of 59 patients were analyzed for *ABCA4* mutations using the ABCR400 microarray and PCR-based sequencing of all coding exons and flanking intronic regions. MLPA analysis as well as sequencing of two regions in introns 30 and 36 reported earlier to harbor deep intronic disease-associated variants was used in 4 selected cases.

**Results:**

An overall detection rate of at least one mutant allele was achieved in 52 of the 59 patients (88.1%). Direct sequencing improved significantly the complete characterization rate, that is, identification of two mutations compared to the microarray analysis (93.1% versus 50%). In total, 40 distinct potentially disease-causing variants of the *ABCA4* gene were detected, including six previously unreported potentially pathogenic variants. Among the disease-causing variants, in this cohort, the most frequent was c.5714+5G>A representing 16.1%, while p.Gly1961Glu and p.Leu541Pro represented 15.2% and 8.5%, respectively.

**Conclusions:**

By using a combination of methods, we completely molecularly diagnosed 48 of the 59 patients studied. In addition, we identified six previously unreported, potentially pathogenic *ABCA4* mutations.

## 1. Introduction

Stargardt disease (STGD1, OMIM 248200) is a juvenile-onset form of macular dystrophy (MD) [1]. It is inherited mainly in an autosomal recessive manner and is one of the most frequent causes of MD in childhood [2]. The disease is characterized by loss of photoreceptor cells in the macula and affects 1 in 10,000 individuals [3]. STGD1 is associated with the appearance of orange-yellow flecks around the macula and accumulation of lipofuscin—a combination of fluorescent by-products of the visual cycle—in the choroid. This accumulation appears to have toxic effects on the retinal pigment epithelium [4].

Mutations in the *ABCA4* (gene ID: 24; OMIM: 601691) gene have been described in *ABCA4*-associated retinopathies, including STGD1, cone-rod dystrophy (CRD), retinitis pigmentosa (RP), and retinal dystrophy [1, 5]. *ABCA4* is a large gene that consists of 50 exons located in chromosome 1p13. It is a member of the subfamily A of the ATP-binding cassette (ABC) transporters that is expressed in the retinal photoreceptors. This gene is involved in the transport and clearance of all-transretinal aldehyde, a by-product of the retinoid cycle of vision, and other essential molecules across the disc membrane into the cytoplasm [6–8]. It is the only gene whose mutations are known to be associated with autosomal recessive STGD1 [3]. Over 800 disease-causing mutations have been identified to date in *ABCA4*-associated phenotypes. The *ABCA4* mutation spectrum ranges from single base substitutions to deletions of several exons, although the majority of reported changes are missense mutations [9–11]. Numerous genetic studies on STGD1 patients have revealed that the disease-associated *ABCA4* alleles are extraordinarily heterogeneous. While some founder mutations accounting for a significant proportion of disease alleles have been identified in specific ethnic groups, hundreds of extremely rare or private variants have also been identified [12–14].

In this study, we used a combination of the ABCR400 microarray analysis and direct sequencing of the entire coding sequence of *ABCA4* gene to investigate the frequency and pattern of disease-associated mutations and polymorphisms in a large cohort of Greek patients with presumed STGD1. Our data extend the mutational spectrum of the *ABCA4* gene and, in addition, facilitate the screening of *ABCA4* mutations in Greek patients by identifying a set of three prevalent alleles.

## 2. Methods

This study adhered to the tenets of the Declaration of Helsinki and the ARVO statement of human subjects and was approved by the Human Subjects Review Committee at the University Hospital of Heraklion, Crete. Informed written consent was obtained for all study participants.

All patients were referred for genotyping after a clinical diagnosis of presumed Stargardt disease was put from treating ophthalmologists. A complete medical and familial history (including consanguineous marriages) was obtained from each patient. Age of symptom onset was documented. Familial STGD1 cases had to be compatible with autosomal recessive inheritance.

### 2.1. *Mutation Screening of the ABCA4 Gene*

Total genomic DNA was extracted from whole blood samples on an iPrep purification instrument using the iPrep PureLink gDNA Blood Kit (Invitrogen, Life Technologies, Carlsbad, CA) according to the manufacturer's instructions. A group of 30 Greek STGD1 patients were analyzed for variants on the ABCR400 microarray version 11.0 including 513–558 alterations (http://www.asperbio.com), as described elsewhere [15]. A second independent group of 29 patients and 8 patients from the microarray group were analyzed by direct sequencing of all 50 exons and intron-exon junctions of the *ABCA4* gene following PCR amplification with PCR primers designed using the Web Primer program. To test for deep intronic sequence variants [16] in 4 patients, primer pairs *ABCA4*-Exon30.1 fwd (5′-CACCCGATCCCTGCTCTTTTTTAT-3′)/rev (5′-TTACATTTTGTCCAGGGACCAAGG-3′) and *ABCA4*-Exon36.1 fwd (5′-ACAGGGATCATTATGACATCAACCCC-3′)/rev (5′-AGCTACATCTCTCTCCATAGGCTCAGA-3′) were used to amplify and sequence a 406- and 596-bp fragment within *ABCA4* intervening sequences 30 and 36, respectively. All primer sequences are shown in Supplementary Table 1. We followed the methods of Kamakari et al. [17] for PCR and Sanger sequencing conditions. Specifically, PCR reactions were performed in a 25 *μ*l total reaction volume, containing 50–100 ng genomic DNA, 2.5 *μ*l of 10x PCR buffer (w/o MgCl_2_), 3 *μ*l of 10 mM dNTPs mix, 0.75 *μ*l of 50 mM MgCl_2_, 1.75 *μ*l of 10 *μ*Μ forward primer, 1.75 *μ*l of 10 *μ*Μ reverse primer, and 0.25 *μ*l of 5 U/*μ*l Platinum Taq DNA polymerase (Invitrogen, Life Technologies). Amplification was performed with the following cycling profile: incubation at 94°C for 5 min followed by 36 cycles of 45 sec denaturation at 94°C, 45 sec annealing at 58°C, and 45 sec elongation at 72°C. The last cycle was followed by a final extension of 3 min at 72°C. Excess primers and dNTPs were removed using exonuclease I and shrimp alkaline phosphatase, and PCR products were sequenced with the BigDye Terminator v3.1 Cycle Sequencing Kit (Applied Biosystems, Foster City, CA) according to the manufacturer's instructions. All sequences were analyzed in forward and reverse directions on an ABI3500 fluorescent sequencer (Applied Biosystems). Nucleotide sequences were compared with the published DNA sequence of *ABCA4* gene (GenBank accession number NG_011605.1 or NG_009073.1) and cDNA (GenBank accession number NM_000350.2). For the *ABCA4* gene, cDNA numbering +1 corresponds to A in the ATG translation initiation codon of ABCA4 transcript.

### 2.2. Multiplex Ligation-Dependent Probe Amplification

Multiplex ligation-dependent probe amplification (MLPA) reagents were obtained from MRC-Holland (SALSA MLPA kit P151-P152 ABCA4, Amsterdam), and the reactions were performed according to the manufacturer's instructions (MRC-Holland).

Whenever possible, segregation analysis was performed according to whether relatives were willing to offer clinical material.

## 3. Results

A total of 59 Greek patients with a presumed clinical diagnosis of STGD1 were screened for *ABCA4* mutations, including 25 males and 34 females. At least two cases reported consanguinity, and notably, two other cases had affected relatives across two successive generations which represent an atypical pattern of inheritance for the disease. The patients are presented in two groups. The first group of 30 patients was screened by an early version of the ABCR400 microarray chip (version 11.0) and the second group of 29 patients by direct sequencing. Direct sequencing was also performed as a complementary method in a subset of 8 patients of the microarray group with one identified mutation leading to the identification of the second mutant allele in 6 of them (Supplementary Table 2). Finally, additional sequencing of two regions in introns 30 and 36 as well as MLPA analysis in 4 patients with one single mutation from both subgroups did not identify the second mutant allele.

ABCR400 *Microarray Screening*. Of the 30 patients screened using the ABCR400 microarray, 15 (50%) were found to carry two disease-causing mutations whereas 8 were found to carry a single mutation in heterozygous state (26.7%) (Supplementary Table 2). No mutations were identified in 7 patients (23.3%).

*Direct Sequencing and MLPA Analysis*. Of the 29 patients screened by PCR-based Sanger sequencing, 27 (93.1%) were found to carry two disease-causing mutations whereas 2 carried a single mutation (6.9%) (Supplementary Table 2). In addition, direct sequencing was performed in the 8 patients of the microarray-analyzed group with one identified mutation leading to the identification of the second mutation in 6 of them. Conclusively, 4 patients from both subgroups remained with one identified mutation. These 4 patients were tested for the known pathogenic deep intronic mutations in introns 30 and 36 [16] and, in addition, screened for large deletions or duplications by MLPA analysis, but no second mutation was identified in them.

In addition, direct sequencing revealed several polymorphic variants in *ABCA4* gene summarized in Supplementary Table 3.

### 3.1. Frequency of Mutations

Altogether, 100 potentially disease-causing *ABCA4* alleles were identified in this cohort of 59 Greek patients presenting with presumed STGD1. Among these 100 mutant alleles, a total of 40 distinct mutations were identified (Table 1). However, three mutations (c.5714+5G>A, p.Gly1961Glu, and p.Leu541Pro (alone or as a complex allele with the p.Ala1038Val)) occurred at a significantly higher frequency, compared to other variants in this cohort (Supplementary Table 2, Table 1). The most frequent mutation c.5714+5G>A (intron 40) was detected in 19 alleles in 16 patients (13 heterozygous, 3 homozygous) accounting for 16.1% of the total number of alleles (*n* = 118). The second most frequent mutation p.Gly1961Glu was detected in 18 alleles in 18 patients (all heterozygous) accounting for 15.2% of the total number of alleles (*n* = 118). The third most frequent mutation p.Leu541Pro was detected in 15 alleles either as a single mutation (5 alleles) or as a complex allele with the p.Ala1038Val (10 alleles) in 14 patients (13 heterozygous, 1 homozygous) accounting for 12.7% (*n* = 118). p.Ala1038Val was not detected as a single allele (Supplementary Table 2, Table 1).

A few mutations were detected more than once, that is, p.Gly607Arg in 4 alleles in 4 patients (all heterozygous), accounting for 3.3% (*n* = 118), and p.Val1973^∗^ and the novel mutation c.5714+1G>C (intron 40) both found in 3 alleles in 2 patients each (1 heterozygous, 1 homozygous), each accounting for 2.45% (*n* = 118). In addition, mutations c.4352+1G>A (intron 29) first reported by us [18], p.Arg18Trp, p.Arg212Cys, and p.Arg1108Cys were detected each in 2 alleles in 4 patients (all heterozygous), each accounting for 1.6% (*n* = 118). In addition, the allelic variant p.Arg1108Leu of the latter mutation was found in 1 patient.

The remainder of 22 distinct mutations was detected once in heterozygosity each accounting for 0.8% (*n* = 118) (Supplementary Table 2, Table 1), including five of the six previously unreported potentially pathogenic mutations p.Trp12^∗^ (exon 1), p.Asn76Thr (exon 3), p.Ser673Argfs^∗^6 (exon 14), p.Cys698Arg (exon 14), and c.4352+4A>C (intron 29). Mutations p.Asn76Thr and p.Cys698Arg and the previously reported p.Arg107^∗^, p.Pro143Leu, p.Gly607Arg, and p.Trp1449^∗^ were detected in compound heterozygosity with the most frequent mutation c.5714+5G>A in intron 40. Mutations p.Arg290Trp, p.Gly607Arg, p.Ser673Argfs^∗^6, c.4352+1G>A, p.Glu1087Lys, p.Arg1108Cys, p.Met1115Cysfs^∗^33, p.Glu1271Gly, p.Gln1412^∗^, p.Cys1488Arg, p.Ser1696Asn, c.5714+1G>C, p.Val1973^∗^, and p.Arg2038Trp were detected in compound heterozygosity with the second most frequent mutation p.Gly1961Glu. Mutations p.Gly2146Asp and p.Arg1108Leu were detected in compound heterozygosity with c.4352+1G>A (intron 29) and p.Arg212Cys, respectively. Some of these once occurring mutations were detected in the same patient, namely, p.Arg18Trp and p.His1625Gln (patient ABCA4-20A), p.Glu1122Lys and p.Gly1591Arg (patient ABCA4-39A), and p.Arg220Cys and p.Gly607Arg (patient ABCA4-35A).

In 4 patients, only one mutant allele was identified even after the use of direct sequencing and MLPA, these mutant alleles being p.Asn380Lys, p.Arg1640Trp, and c.5714+5G>A (the latter in 2 patients).

The majority of the distinct mutations were evenly distributed along the exons of the *ABCA4* gene. Three different mutations were observed in exon 22 and two in each of exons 1, 4, 6, 13, 14, 23, 35, and 44 and introns 29 and 40; however, no accumulation in a specific exon was observed. No mutation was identified in exons 2, 5, 7, 10, 11, 15, 18–20, 24, 26, 27, 31, 32, 34, 37–41, 45, 46, and 48–50 which comprise 50% (25/50) of the exons of the *ABCA4* gene.

Twenty-six of the detected mutations were missense, 5 were splice defects, 6 were nonsense, and 3 were frameshift mutations (Table 1).

### 3.2. Novel Mutations

Six novel mutations, namely, p.Trp12^∗^ (exon 1), p.Asn76Thr (exon 3), p.Ser673Argfs^∗^6 (exon 14), p.Cys698Arg (exon 14), c.4352+4A>C (intron 29) and c.5714+1G>C (intron 40), and c.4352+1G>A (intron 29) previously reported by us [18], were detected in this study. Two were missense, three were splicing mutations (two of which occur in intron 29), one nonsense, and one small deletion of 13 nucleotides leading to a frameshift mutation of the *ABCA4* gene. These seven distinct mutations were detected in 10 apparently unrelated patients, 9 in heterozygous, and 1 in homozygous state. p.Trp12^∗^ (exon 1), p.Asn76Thr (exon 3), p.Ser673Argfs^∗^6 (exon 14), p.Cys698Arg (exon 14), and c.4352+4A>C (intron 29) were each detected once in heterozygosity in 5 respective patients, and c.5714+1G>C was detected twice in two patients (1 in homozygosity, 1 in heterozygosity) and c.4352+1G>A (in three patients in heterozygosity).

Of these, the mutations p.Asn76Thr and p.Cys698Arg were detected in compound heterozygosity with the most frequently detected mutation c.5714+5G>A (intron 40) in one male (ABCA4-47A, Supplementary Figures 1A and B) and one female patient (ABCA4-27A, Supplementary Figures 2A and B), respectively. The age of onset for patient ABCA4-47A carrying the p.Asn76Thr was 52 years according to the patient who was initially erroneously diagnosed with age-related macular degeneration in 2012. He had a cataract surgery at the age of 42 and eventually diagnosed with presumed Stargardt disease of late onset.

The age of onset for patient ABCA4-27A carrying the novel potentially pathogenic missense mutation p.Cys698Arg was 13 years of age.

Mutation c.4352+4A>C (intron 29) was detected in 1 patient in compound heterozygosity with p.Arg18Trp (exon 1) (patient ABCA4-37A, Supplementary Figures 3A and 3B). The age at diagnosis for this patient was 15. Segregation analysis documented that the unaffected parents were heterozygous for each mutation with the c.4352+4A>C paternally inherited and the p. Arg18Trp maternally inherited whereas her affected sister carried both mutations (Supplementary Figure 3A).

c.4352+1G>A was detected in two unrelated patients in compound heterozygosity with p.Gly2146Asp (exon 47) in patient ABCA4-5A (Supplementary Figures 4A and 4B) and with the complex allele p.Leu541Pro/p.Ala1038Val (exons 12/21) in patient ATH44A, (Supplementary Figures 5A and B). For patient ATH44A, five additional family members including two affected siblings, their unaffected parents, and an unaffected maternal uncle were tested for the two mutations. Both affected siblings were compound heterozygous for the two mutations; the mother was a heterozygous carrier of the c.4352+1G>A and the father of the p.Leu541Pro/p.Ala1038Val (Supplementary Figure 5A) whereas the maternal uncle was free of mutation.

The c.5714+1G>C mutation was initially detected in homozygous state in one young female patient (ABCA4-31A, Supplementary Figures 6A–C). Five additional family members including her unaffected parents (ABCA4-31B, C), her two affected maternal third male cousins (ABCA4-33A, B), and their unaffected mother (ABCA4-33C) were tested for the novel mutation. All parents were found heterozygous carriers whereas the affected cousins were homozygous (Supplementary Figures 6A–6C). All three young patients were diagnosed with presumed Stargardt disease approximately at the age of 7. Furthermore, the same novel potentially pathogenic mutation was detected in a seemingly unrelated patient (ABCA4-14A, Supplementary Figures 7A and 7B) in compound heterozygosity with the p.Gly1961Glu mutation.

Genetic analysis of patient (ATH73A) revealed a novel nonsense mutation c.36G>A (p.Trp12^∗^) and a previously reported mutation c.571-2A>T occurring in a compound heterozygous state in exon 1 and intron 5, respectively, in the ABCA4 gene (Supplementary Figures 8A and 8B). This mutation leads to premature truncation after just 12 amino acids, which is likely to render the protein to be nonfunctional or lead to nonsense-mediated mRNA decay. It is included in the SNP database (https://www.ncbi.nlm.nih.gov/snp/) (rs761209432); however, according to our knowledge, it has not been associated previously with disease.

The sixth mutation c.2019_2031delCATCGTCTTGGAG was detected in patient ATH-53A. It consists of a deletion of 13 nucleotides from c.2019 to c.2031 (Supplementary Figures 9A and 9B) which would cause a frameshift that changes codon Ser673 to Arg, leading to a premature stop codon at position 678 (p.Ser673Argfs^∗^6). The predicted translation product from this allele would consist of 677 amino acid residues instead of the 2273 found in the mature protein. This mutation was detected in compound heterozygosity with the p.Gly1961Glu mutation. The disease onset for this patient was at the age of 17.

In silico analysis for missense variants using the predictive algorithms of “Sorting Intolerant From Tolerant” ((SIFT) in the public domain, http://sift.jcvi.org), Polymorphism Phenotyping v2 ((PolyPhen-2) in the public domain, http://genetics.bwh.harvard.edu/pph2), and Protein Variation Effect Analyzer ((PROVEAN) in the public domain, http://provean.jcvi.org/index.php) tools predicted novel changes p.Asn76Thr and p.Cys698Arg to have a deleterious effect, whereas novel changes c.4352+4A>C and c.5714+1G>C are predicted to affect normal splicing by the Human Splicing Finder ((HSF); in the public domain, http://www.umd.be/HSF3/ prediction tool (Supplementary Table 4)).

### 3.3. Results of Relatives

Allelic segregation analyses were performed in 11 families. A total of 37 relatives were tested (data not shown). The analysis of one family (F11) that showed atypical pattern of inheritance is presented in detail hereafter.

### 3.4. Family F11A: A Family with an Atypical Pattern of Inheritance

**Figure 1:**
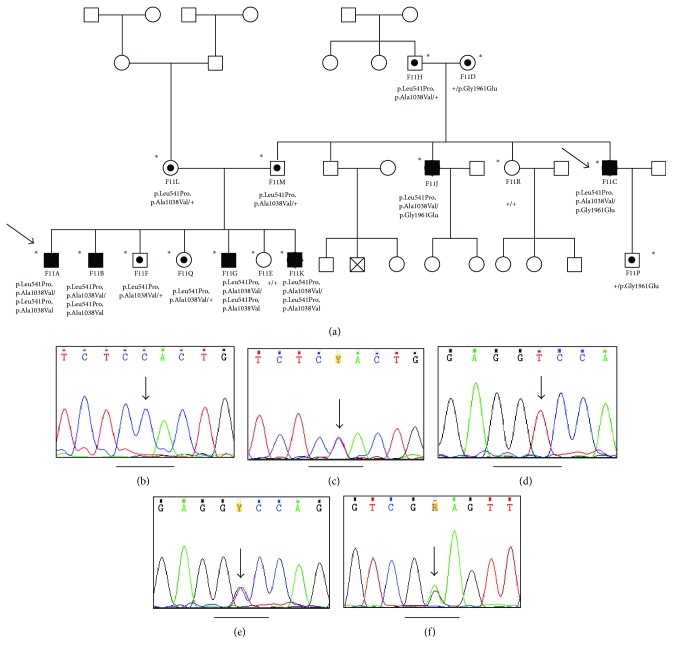
Family F11. (a) Pedigree of patients F11A and F11C (probands). Arrows indicate the probands. Affected and unaffected individuals are represented with black and open circles, respectively. Males are represented with a quadrant and females with a circle. The asterisk denotes the individuals genetically examined. A dot in a symbol denotes an unaffected carrier of the respective mutation. Pluses (+) denote the wild type alleles. (b, c, d, and e) Sequence chromatograms of the F11A and F11C showing the complex allele c.1622T>C/c.3113C>T (p.Leu541Pro/p.Ala1038Val) in homozygosity in proband F11A (b, d) and in heterozygosity in proband F11C (c, e). (f) Sequence chromatogram of the proband F11C showing the missense mutation c.5882G>A (p.Gly1961Glu) in heterozygosity on the second allele. The arrow indicates the position of the G to A substitution at the second nucleotide of codon 1961 encoded by GGA marked by a horizontal line.

In this family, the male proband (F11A, Figure 1(a)) and one of his brothers (F11B, Figure 1(a)) were diagnosed with presumed STGD1 before school age. Their parents (F11M, F11L) were unaffected which supported an autosomal recessive pattern of inheritance. However, two paternal aunts (F11C and F11J) were also affected but the disease presented in their twenties. Genotyping using the ABCR400 microarray was performed initially in the proband (F11A) and in one of his two affected aunts (F11C). The analysis revealed the complex p.Leu541Pro/p.Ala1038Val mutation in both of them. The proband was homozygous for the detected mutation; however, his aunt was compound heterozygous with p.Gly1961Glu. Following the initial analysis, thirteen additional family members including the proband's six siblings, parents, paternal grandparents, paternal aunts, and paternal cousin were tested for the two aforementioned mutations. Their results shown in Figures 1(a)–1(f) confirmed that the proband's affected siblings presenting with an early onset severe form of the disease were homozygous for the p.Leu541Pro/p.Ala1038Val complex allele whereas his second affected aunt with the later onset milder phenotype was a compound heterozygote with the p.Gly1961Glu mutation. The proband's unaffected parents were found heterozygous carriers for the p.Leu541Pro/p.Ala1038Val mutation. In addition, the paternal grandmother was a heterozygous carrier of the p.Gly1961Glu mutation and his grandfather heterozygous for the p.Leu541Pro/p.Ala1038Val. The parents of the proband did not report consanguinity, but their ascendants originated from the same part of Northern Greece. Genotyping resolved the atypical pattern of inheritance of STGD1 disease in this family.

## 4. Discussion

Genetic analysis of 59 Greek patients with presumed STGD1 revealed 40 different mutations in the *ABCA4* gene and an overall detection rate of at least one mutant allele in 88.1% (52 patients) using a combination of the ABCR400 microarray, direct sequencing, and MLPA methods. Of these 52 patients, 48 were completely characterized (two mutant alleles detected) (81.3%) and 4 partially characterized (one mutant allele detected) (6.7%). This is well within the published range of biallelic mutation identification that ranges between 55% and 80% [19]. However, clinical heterogeneity and variable diagnostic criteria in our cohort of patients who were referred by a number of collaborating ophthalmologists may have led to uncertainties in clinical diagnosis in some of our patients.

Specifically, using the ABCR400 microarray screening method between the years 2006 and 2009 in 30 patients, two mutations were identified in 15 (50%), one mutation in 8 (26.7%), and no mutation in the remaining 7 (23.3%) cases. Valverde et al. [15] have reported similar mutation detection rates using the same microarray in a cohort of 76 patients, but Jaakson et al. [13] have reported somewhat lower detection rates (36.6% with two mutations and 24.4% with no mutations).

Using direct sequencing in 29 patients, both disease-causing mutations were identified in 27 (93.1%) whereas one mutation was identified in 2 patients (6.9%). Moreover, direct sequencing of *ABCA4*, used as a complementary method in 8 patients previously found to harbor one mutant allele using the microarray method of screening, led to the identification of a second mutant allele in 6 of them thus raising the complete characterization rate (identification of both mutant alleles) to 70% in this group. Overall, the use of direct sequencing as an initial screening method led to significantly higher complete characterization rate when compared to the microarray (93.1% versus 50%, resp.); therefore, it became the method of choice for *ABCA4* mutation screening by our group. These rates of detection parallel well with the recently published literature [20–22]. For example, in the study by Riveiro-Alvarez et al. [21], the mutation detection rate after complete *ABCA4* sequencing with Sanger sequencing or next generation sequencing (NGS) was 73.6% in arSTGD1 patients.

Most of the patients (42; *n* = 59) (71.1%) were compound heterozygotes. Only six patients were found to be homozygotes (Table 2). A single disease allele was found in 6.7% (4/59) of our cases. No disease allele was detected in 11.8% (7; *n* = 59) of our patients. Possible explanations for this may include erroneous clinical diagnosis, as well as the presence of disease-causing mutations in parts of the gene that were not screened, for example, deep into the introns or other regulatory regions and the presence of disease-causing mutations in other genes. The latter has proved to be the case in two of our ABCA4 negative patients which were found to harbor mutations in CRX and PROM1 genes (unpublished data).

Concurring with earlier studies [10, 23, 24], most *ABCA4* mutations are distributed throughout the entire coding sequence, and no mutational hotspots seem to exist. However, it is of note that 4 mutations lie in introns 29 and 40 (2 in each), 3 in exon 22 and 2 each in exons 1, 4, 6, 13, 14, 23, 35, and 44. In addition, mutations have been detected in half of the exons of the *ABCA4* gene in this cohort; therefore, perhaps initial *ABCA4* screening of Greek patients could include only this set of exons.

Most of the detected mutations were missense mutations (26), 5 were splice defects, 6 were nonsense, and 3 were frameshift mutations (Table 1).

A prevalent set of three mutant alleles composed of c.5714+5G>A representing 16.1% of all screened alleles (19/118), p.Gly1961Glu representing 15.2% (18/118), and p.Leu541Pro either as a single mutation or as a complex allele with the p.Ala1038Val representing 12.7% (15/118) was identified. Notably, of the 48 completely characterized subjects, 13 had two mutated alleles and 24 had one mutated allele from this high incidence set. Therefore, based on the mutation frequencies observed, one would expect that screening for these three mutations only would lead to complete genetic characterization of approximately 22% (13; *n* = 59) or partial genetic characterization of approximately 66.1% (39; *n* = 59) of Greek STGD1 patients providing strong indication of a true *ABCA4*-related retinopathy. Specifically, in our cohort, the most frequent mutation is the intronic splicing mutation IVS40+5G>A, accounting for 16.1% of all screened alleles (19/118). This is considerably higher than the frequency observed in other Europeans (3.9% in Spanish, 4.2% in Italian, and 8.6% in Hungarian patients) [15, 23, 24]. This mutation confers a milder phenotype, and this is especially true when the mutation is in homozygosity (3 patients in our cohort, H1, ATH35A, and ABCA4-44A) with an age of onset at the 4th decade; these patients consist half of the homozygotes in this cohort. The second most frequent mutation is p.Gly1961Glu with a frequency of 15.2% in our cohort of patients. This mutation is one of the most frequently mutated alleles in several populations analyzed. Its frequency ranges from 9% to 9.5% in some European countries or even lower in Spanish and Portuguese patients to 20-21% in Italian, Slovenian, and German patients. Its frequency of 15.2% in our cohort is comparable with the average frequency of 10% for the central European populations. The third most frequent mutation in Greek patients is p.Leu541Pro, alone or as a complex with p.Ala1038Val, with a frequency of 12.7% (15/118). This mutation is traditionally considered to be a German allele and is found in Germanic populations in general with a very low representation in other European populations, for example, 0.7% of the mutated alleles in an Italian cohort [25]. However, in our Greek cohort of patients, this is one of the three prevalent alleles. Although difficult to interpret, this high prevalence of the particular allele among Greek patients may be due to the fact that there has been a strong interaction and tight bonds between Greeks and Germans throughout the years with significant immigration from Greece to Germany starting around 1700. Moreover, it is known that in 1828, 31 Bavarian families came to Greece along with King Otto of Greece (http://www.greece-is.com/little-bavaria-in-athens/) and were settled in Greece and their Hellenized offspring still living in Greece today. Moreover, it is estimated that between the 1960s and 1970s, 500,000 Greek young people migrated and approximately 30% of them returned to Greece. Considering these facts, a phenomenon of gene flow for this prevalent allele through intermarriage cannot be excluded. A similar hypothesis of gene flow has been used to explain the unusually high incidence of b-globin gene mutations in Germans. It has been hypothesized that mutations in two-thirds of the cases have been introduced from the Mediterranean [26, 27].

Six previously unreported potentially pathogenic mutations, namely, p.Trp12^∗^ (exon 1), p.Asn76Thr (exon 3), p.Cys698Arg (exon 14), p.Ser673Argfs^∗^6 (exon 14), c.4352+4A>C (intron 29), and c.5714+1G>C (intron 40) were detected in this study. Their absence from the ExAC (Exome Aggregation Consortium) database and their in silico analysis for the investigation of their pathogenicity, as mentioned in Results, support their potential pathogenicity. In addition, segregating results in the cases of the c.4352+4A>C and c.5714+1G>C splice variants strengthen their potential pathogenicity. Interestingly, the novel splice mutation c.5714+1G>C in intron 40 was detected in two apparently unrelated patients, one male patient from the region of the western Greek islands in the Ionian sea and one female patient from the region of the Cyclades islands (Santorini) in the Aegean sea. It was detected in compound heterozygosity in the first patient and in homozygosity in the second patient. Parents of the second patient were found heterozygous for the mutation thus confirming the true homozygous state of the mutation in the patient. Furthermore, this second female patient who comes from the island of Santorini has two-thirds affected male cousins being siblings between them who were found to bear the same homozygous mutation. This family has other affected relatives across different generations once again proving the not so infrequent atypical pattern of inheritance in STGD1 families [28]. The family living in the same village of the island shared the information with us that there are many similarly affected patients in their village. We presume that they all bear the same homozygous mutation due to their common origin and endogamy, and we intend to offer a simple targeted testing to them per their request. It is of note that the paternal origin of the patient from the western Greek islands is from Santorini, and it would be interesting to verify that the c.5714+1G>C is his paternal allele, that is, the same which was detected in the patients from Santorini.

In addition, mutation c.4352+1G>A that was first described by our group in 2009 [18] was detected in two unrelated patients in our cohort in compound heterozygosity with p.Gly2146 (exon 47) (patient ABCA4-5A) or in conjunction with the complex allele p.Leu541Pro/p.Ala1038Val (exons 12/21) (patient ATH44A).

One family (F-F11) presented with an atypical pattern of inheritance of STGD1 with affected members of different age of onset in two successive generations. The male proband (F11A) and his brother (F11B) were diagnosed with presumed STGD1 before school age, whereas their parents were unaffected, and two paternal aunts (F11C and F11J) were diagnosed with the disease in their late twenties. Genotyping of the proband and one of his affected aunts revealed that the proband was homozygous for the p.Leu541Pro/p.Ala1038Val, whereas his aunt was a compound heterozygote with the p.Gly1961Glu mutation. 13 additional family members were tested for these two mutations as described in Results. In summary, this family segregates two different pathogenic *ABCA4* variants. Based on the different age of onset of the disease in the proband and his other affected siblings and his affected aunts, we assume that the milder phenotype of the two affected aunts is likely due to the p.Gly1961Glu which has been previously associated with a late-onset, milder disease phenotype [28, 29]. Due to the high carrier frequency of disease-causing *ABCA4* alleles in the general population (1 : 20), pseudodominant inheritance in STGD1 has been described [9, 30–32].

All patients of this cohort were referred for genotyping from a network of collaborating ophthalmologists after a clinical diagnosis of presumed Stargardt was put. However, details of the phenotype were not always available thus precluding a systemic genotype-phenotype correlation. Future work towards this direction could provide additional helpful clinical data.

## 5. Conclusions

In summary, we report here the mutational spectrum of the *ABCA4* gene in a large Greek cohort, with successful identification of both disease-causing alleles at a rate of 81.3% (48/59). In addition, we report the identification of six novel potentially pathogenic mutations thus extending the spectrum of the *ABCA4* gene mutations and of three prevalent alleles which may facilitate the screening of Greek patients with *ABCA4*-related retinopathy. The complete genetic characterization of the patients will improve the accuracy of diagnosis and their counselling and also will assist in more effective patient selection of genetically confirmed participants for current and future clinical trials for *ABCA4*-associated retinal diseases.

## Figures and Tables

**Table 1:** Frequency of 40 distinct mutations detected in 59 Greek patients with presumed STGD1.

Mutation number	Amino acid change	Nucleotide change	Exon	Number of alleles detected	Frequency	References
1	p.Trp12^∗^	c.36G>A	1	1	0.8%	This study
2	p.Arg18Trp	c.52C>T	1	2	1.6%	[33]
3	p.Asn76Thr	c.227A>C	3	1	0.8%	This study
4	p.Pro143Leu	c.428C>T	4	1	0.8%	[13]
5	p.Arg107^∗^	c.319C>T	4	1	0.8%	[34]
6	NA	c.571-2Α>Τ	Intron 5	1	0.8%	[35]
7	p.Arg212Cys	c.635C>T	6	2	1.6%	[33]
8	p.Arg220Cys	c.658C>T	6	1	0.8%	[11]
9	p.Arg290Trp	c.868C>T	8	1	0.8%	[36]
10	p.Asn380Lys	c.1140T>A	9	1	0.8%	[11]
11^a^	p.Leu541Pro	c.1622T>C	12	10	8.5%	[37]
12	p.Gly607Arg	c.1819G>A	13	4	3.3%	[10]
13	p.Asp645Asn	c.1933G>A	13	1	0.8%	[9]
14	p.Ser673Argfs^∗^6	c.2019_2031del13	14	1	0.8%	This study
15	p.Cys698Arg	c.2092T>C	14	1	0.8%	This study
16	p.Ser795Argfs^∗^43	c.2385_2400delCTTACTGTCTCCGGTG	16	1	0.8%	[38]
17	p.Gln876^∗^	c.2626C>T	17	1	0.8%	[39]
18	p.Ala1038Val	c.3113C>T	21	6	5.1%	[3]
19	p.Arg1108Cys	c.3322C>T	22	2	1.6%	[37]
20	p.Arg1108Leu	c.3323G>T	22	1	0.8%	[36]
21	p.Glu1087Lys	c.3259G>A	22	1	0.8%	[3]
22	p.Met1115Cysfs^∗^33	c.3342delC	23	1	0.8%	[36]
23	p.Glu1122Lys	c.3364G>A	23	1	0.8%	[9]
24	p.Glu1271Gly	c.3812A>G	25	1	0.8%	[40]
25	p.Gln1412^∗^	c.4234C>T	28	1	0.8%	[12]
26	p.Trp1449^∗^	c.4346G>A	29	1	0.8%	[9]
27	NA	c.4352+1G>A	Intron 29	3	2.5%	[18, 40, 41]
28	NA	c.4352+4A>C	Intron 29	1	0.8%	This study
29	p.Cys1488Arg	c.4462T>C	30	1	0.8%	[9]
30	p.Gly1591Arg	c.4771G>A	33	1	0.8%	[42]
31	p.Arg1640Trp	c.4918C>T	35	1	0.8%	[37]
32	p.His1625Gln	c.4875T>A	35	1	0.8%	[13]
33	p.Ser1696Asn	c.5087G>A	36	1	0.8%	[9]
34	NA	c.5714+5G>A	Intron 40	19	16.1%	[30]
35	NA	c.5714+1G>C	Intron 40	3	2.5%	This study
36	p.Gly1961Glu	c.5882G>A	42	18	15.2%	[43]
37	p.Val1973^∗^	c.5917delG	43	3	2.5%	[10]
38	p.Leu2026Pro	c.6077T>C	44	1	0.8%	[44]
39	p.Arg2038Trp	c.6112C>T	44	1	0.8%	[3]
40	p.Gly2146Asp	c.6437G>A	47	1	0.8%	[36]

^a^4 times was detected as single and 6 times as complex with p.A1038V.

## Supplementary Materials

Supplementary Materials Supplementary Figure 1: family ABCA4-47. Supplementary Figure 2: family ABCA4-27. Supplementary Figure 3: family ABCA4-37. Supplementary Figure 4: family ABCA4-5. Supplementary Figure 5: F-ATH44. Supplementary Figure 6: F-ABCA4-31/33. Supplementary Figure 7: F-ABCA4-31/33. Supplementary Figure 8: F-ATH73. Supplementary Figure 9: F-ATH53. Supplementary Table 1: *ABCA4* PCR primer sequences. Supplementary Table 2: mutations detected in the ABCA4 gene in a cohort of 59 Greek patients with presumed STGD1. Supplementary Table 3: polymorphic variants of the ABCA4 gene detected in Greek STGD patients. Supplementary Table 4: results of in silico analysis for 2 novel missense variants using the predictive algorithms SIFT, PolyPhen-2, and PROVEAN and for 2 novel splice variants using HSF prediction tool.
