# Corrigendum to “Mutation Spectrum of the ABCA4 Gene in a Greek Cohort with Stargardt Disease: Identification of Novel Mutations and Evidence of Three Prevalent Mutated Alleles”

**DOI:** 10.1155/2018/3039672

**Published:** 2018-12-16

**Authors:** Smaragda Kamakari, Vassiliki Kokkinou, George Koutsodontis, Polixeni Stamatiou, Christoforos Giatzakis, Anastasios Anastasakis, Ioannis Minas Aslanides, Stavrenia Koukoula, Theoni Panagiotoglou, Ioannis Datseris, Miltiadis K. Tsilimbaris

**Affiliations:** ^1^Ophthalmic Genetics Unit, OMMA Ophthalmological Institute of Athens, Athens, Greece; ^2^DNAbiolab, Cretan Center for Research and Development of Applications on Genetics and Molecular Biology, Heraklion, Crete, Greece; ^3^Department of Clinical Electrophysiology of Vision, Athens Eye Hospital, Glyfada, Athens, Greece; ^4^Department of Ophthalmology, Emmetropia Mediterranean Eye Institute, Heraklion, Crete, Greece; ^5^Ophthalmica Institute of Ophthalmology and Microsurgery, Thessaloniki, Greece; ^6^Department of Ophthalmology, University of Crete School of Medicine, Heraklion, Greece; ^7^Department of Retinal Disorders, OMMA Ophthalmological Institute of Athens, Athens, Greece

In the article titled “Mutation Spectrum of the ABCA4 Gene in a Greek Cohort with Stargardt Disease: Identification of Novel Mutations and Evidence of Three Prevalent Mutated Alleles” [1], the first and last names of all the authors were reversed. The revised authors' list is shown above.
