# Supplementary material for: Mutation Spectrum of the ABCA4 Gene in a Greek Cohort with Stargardt Disease: Identification of Novel Mutations and Evidence of Three Prevalent Mutated Alleles
Source: J Ophthalmol. 2018 Apr 30;2018:5706142. doi: 10.1155/2018/5706142 (PMC5952432; doi:10.1155/2018/5706142)
Supplement: Supplementary Materials — Supplementary Figure 1: family ABCA4-47. Supplementary Figure 2: family ABCA4-27. Supplementary Figure 3: family ABCA4-37. Supplementary Figure 4: family ABCA4-5. Supplementary Figure 5: F-ATH44. Supplementary Figure 6: F-ABCA4-31/33. Supplementary Figure 7: F-ABCA4-31/33. Supplementary Figure 8: F-ATH73. Supplementary Figure 9: F-ATH53. Supplementary Table 1: ABCA4 PCR primer sequences. Supplementary Table 2: mutations detected in the ABCA4 gene in a cohort of 59 Greek patients with presumed STGD1. Supplementary Table 3: polymorphic variants of the ABCA4 gene detected in Greek STGD patients. Supplementary Table 4: results of in silico analysis for 2 novel missense variants using the predictive algorithms SIFT, PolyPhen-2, and PROVEAN and for 2 novel splice variants using HSF prediction tool. [file 5706142.f1.docx]

Supplementary Figure 1: F-ABCA4-47

A.

B. c.227A>C (exon 3)


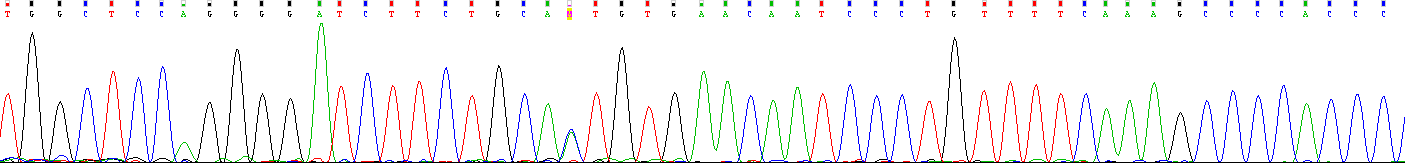


*

Supplementary Figure 1. Family ABCA4-47. A. Pedigree of patient ABCA4-47A (proband). Arrow indicates the proband. Affected and unaffected individuals are represented with black and open circles, respectively. Males are represented with a quadrant and females with a circle. The asterisk denotes the individual(s) genetically examined. B. Sequence chromatogram of the patient ABCA4-47A showing the point mutation c.227A>C (Mut1) leading to the novel p.Asn76Thr missense mutation in heterozygosity. The arrow in B indicates the position of the A to C substitution at the second nucleotide of triplet AAT at codon 76 marked by a horizontal line. This mutation was detected in compound heterozygosity with the previously reported mutation c.5714+5G>A (Mut2).


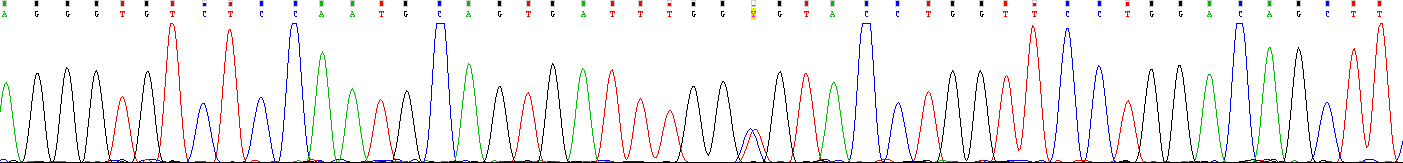


age: 34

age of onset: 14

Mut1:c.2092T>C

Mut1:c.2092T>C

Mut2: c.5714+ 5G>A

Mut2: c.5714+ 5G>A

Mut1/Mut2

Mut1/Mut2

B. c.2092T>C (exon 14)

Supplementary Figure 2: F-ABCA4-27

A.

*

Supplementary Figure 2. Family ABCA4-27. A. Pedigree of patient ABCA4-27A (proband). Arrow indicates the proband. Affected and unaffected individuals are represented with black and open circles, respectively. Males are represented with a quadrant and females with a circle. The asterisk denotes the individual(s) genetically examined. B. Sequence chromatogram of the patient ABCA4-27A showing the point mutation c.2092T>C (Mut1) leading to the novel p.Cys698Arg missense mutation in heterozygosity. The arrow in B indicates the position of the T to C substitution at the first nucleotide of triplet TGT at codon 698 marked by a horizontal line. This mutation was detected in compound heterozygosity with the previously reported mutation c.5714+5G>A (Mut2).

Supplementary Figure 3: F-ABCA4-37

A.

age: 19

age of onset: 15

Mut1: c.52C>T

Mut1: c.52C>T

Mut2: c.4352+4A>C

Mut2: c.4352+4A>C

Mut1/Mut2

Mut1/Mut2

Mut1/Mut2

Mut2/+

Mut1/+

*

*

*

*

B. c.4352+4A>C (intron 29)


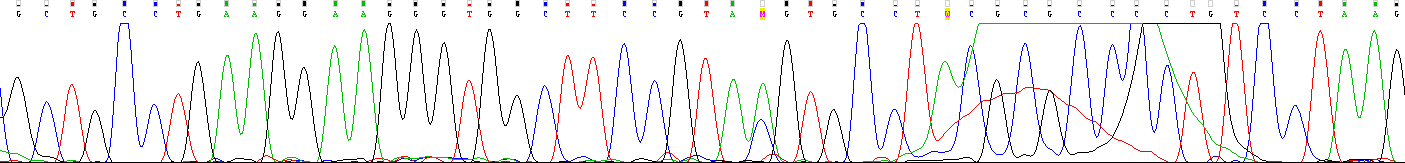


Supplementary Figure 3. Family ABCA4-37. A. Pedigree of patient ABCA4-37A (proband). Arrow indicates the proband. Affected and unaffected individuals are represented with black and open circles, respectively. Males are represented with a quadrant and females with a circle. The asterisk denotes the individual(s) genetically examined. A dot in a symbol denotes an unaffected carrier of the respective mutation. Pluses (+) denote the wild type alleles. B. Sequence chromatogram of the patient showing the novel splicing site mutation c.4352+4A>C (Mut2) in heterozygosity. The arrow indicates the A to C substitution at the forth nucleotide position at the 5′ splice site of intron 29. This mutation was detected in compound heterozygosity with the previously reported point mutation c.52C>T (Mut1) leading to the p.Arg18Trp missense mutation.

Supplementary Figure 4: F-ABCA4-5


Mut2/+

A.

*

*

*

*


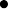


Mut2/+

Mut2/+

Mut2/+

+/+

*

age:18

age of onset:11

+/+

Mut2/+

Mut2/+

B. c.4352+1G>A (intron 29)


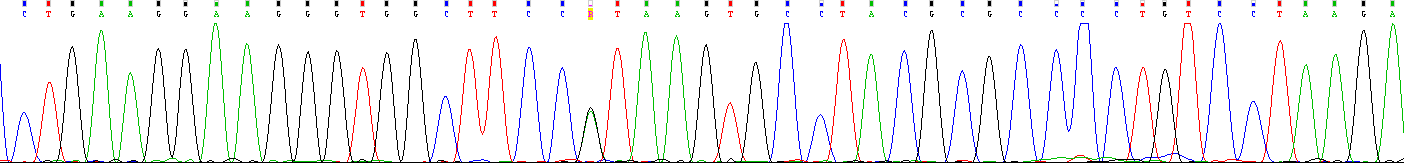


Mut2/+

Supplementary Figure 4. Family ABCA4-5. A. Pedigree of patient ABCA4-5A (proband). Arrow indicates the proband. Affected and unaffected individuals are represented with black and open circles, respectively. Males are represented with a quadrant and females with a circle. The asterisk denotes the individual(s) genetically examined. A dot in a symbol denotes an unaffected carrier of the respective mutation. Pluses (+) denote the wild type alleles. B. Sequence chromatogram of the patient showing the splicing site mutation c. 4352+1G>A (Mut1) in heterozygosity. The arrow indicates the position of the G to A substitution at the 5′ donor splice site of intron 29. This mutation was detected in compound heterozygosity with the previously reported point mutation c.6437G>A (Mut2) leading to the p.Gly2146Asp missense mutation.

Supplementary Figure 5: F-ATH44

A.

*

*

*

*

*

Mut1/Mut2

Mut1/Mut2

age: 25

age of onset: 6

B. c.4352+1G>A (intron 29)


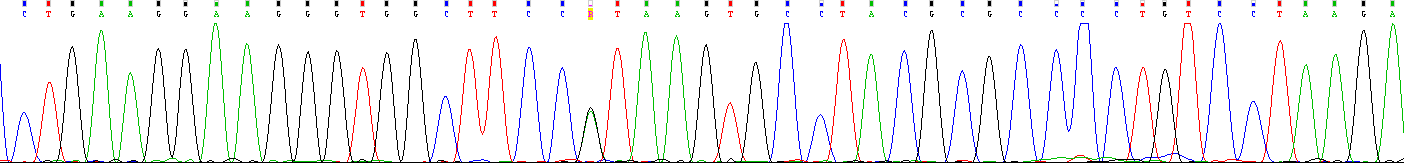


Supplementary Figure 5. Family ATH44. A. Pedigree of patient ATH44A (proband). Arrow indicates the proband. Affected and unaffected individuals are represented with black and open circles, respectively. Males are represented with a quadrant and females with a circle. The asterisk denotes the individual(s) genetically examined. A dot in a symbol denotes an unaffected carrier of the respective mutation. Pluses (+) denote the wild type alleles. B. Sequence chromatogram of the patient showing the splicing site mutation c.4352+1G>A (Mut2) in heterozygosity. The arrow indicates the position of the G to A substitution at the 5′ donor splice site of intron 29. This mutation was detected in compound heterozygosity with the previously reported complex mutation c.1622T>C/c.3113C>T (p.Leu541Pro/p.Ala1038Val.

Supplementary Figure 6: F-ABCA4-31/33

Mut2/+

Mut2/+

Mut1/Mut1

A.

Mut1/+

ABCA4-33C

Mut1/Mut1

ABCA4-33B

ABCA4-33A

Mut1/Mut1

ABCA4-31B

Mut1/+

Mut1/+

ABCA4-31C

ABCA4-31A

Mut1: c.5714+1G>C

*

*

*

*

*

Age:12

Age of onset:7

*

Age:24

Age of onset:7


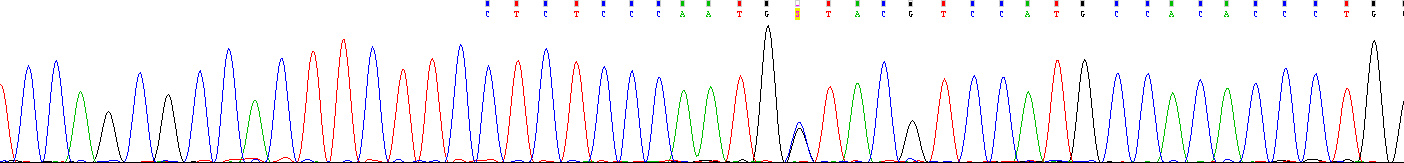


C. c.5714+1G>C (intron 40)

B. c.5714+1G>C (intron 40)


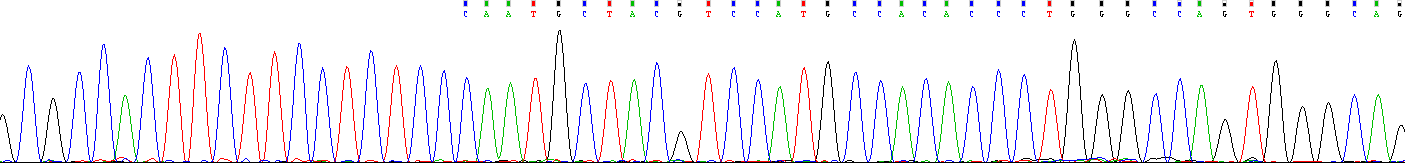


Supplementary Figure 6. DNA sequence analysis of exon 40 and flanking sequences of ABCA4 gene in a family with multiple affected individuals. A. Pedigree of patients ABCA4-31Α and ABCA4-33A related affected individuals (probands). Arrows indicate the probands. Affected and unaffected individuals are represented with black and open circles, respectively. Males are represented with a quadrant and females with a circle. The asterisk denotes the individual(s) genetically examined. A dot in a symbol denotes an unaffected carrier of the respective mutation. Pluses (+) denote the wild type alleles. B. Mutant sequence in proband ABCA4-31A showing the novel splicing site mutation c.5714+1G>C (Mut1) in homozygosity. C. Mutant sequence in proband’s mother ABCA4-31B showing the novel splicing site mutation c.5714+1G>C in heterozygosity. The arrow in B and C indicate the position of the G to C substitution at the 5′ donor splice site of intron 40.

Supplementary Figure 7: F-ABCA4-14


A.


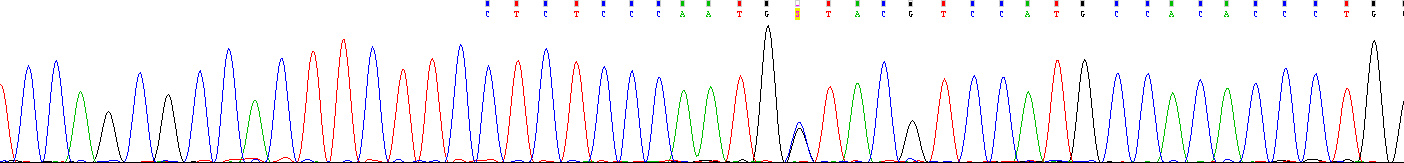


Β. c.5714+1G>C (intron 40)

*

*

*

*

Supplementary Figure 7. Family ABCA4-14. A. Pedigree of patient ABCA4-14A (proband). Arrow indicates the proband. Affected and unaffected individuals are represented with black and open circles, respectively. Males are represented with a quadrant and females with a circle. The asterisk denotes the individual(s) genetically examined. B. Sequence chromatogram of the patient showing the splicing site mutation c.5714+1G>C (Mut1) in heterozygosity. The arrow in B indicates the position of the G to C substitution at the 5′ donor splice site of intron 40. This mutation was detected in compound heterozygosity with the previously reported mutation c.5882G>A (p.Gly1961Glu) (Mut2).

Supplementary Figure 8: F-ATH73

A.

Mut1: p.Trp12*

Mut2: c.571-2A>T

Mut1/Mut2

Mut1/Mut2

*

B. c.36G>A (exon 1)


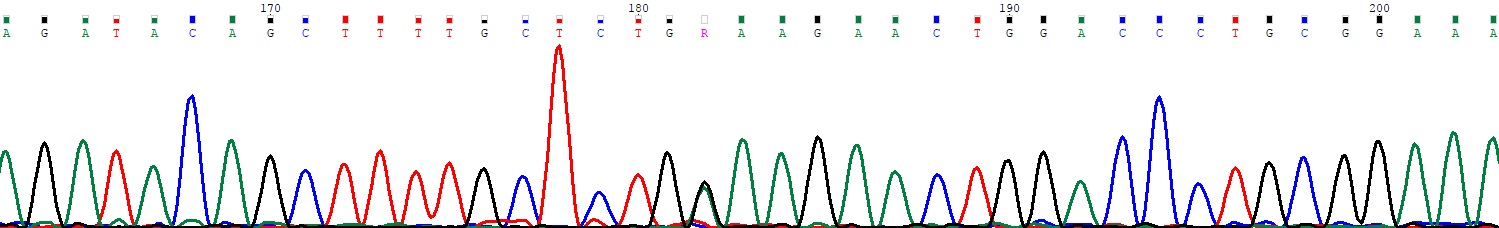


Supplementary Figure 8. Family ATH-73. A. Pedigree of patient ATH-73A (proband). Arrow indicates the proband. Affected and unaffected individuals are represented with black and open circles, respectively. Males are represented with a quadrant and females with a circle. The asterisk denotes the individual(s) genetically examined. B. Sequence chromatogram of the patient showing the novel nonsense mutation c.36G>A (p.Trp12*) in heterozygosity. The arrow in B indicates the position of the G to A substitution at the third nucleotide of triplet TGG at codon 12 marked by a horizontal line. This mutation was detected in compound heterozygosity with the previously reported splicing mutation c.571-2A>T (Mut2).

Supplementary Figure 9: F-ATH53

A.

Mut1: p.Ser673Argfs*6

Mut2: p.Gly1961Glu

age: 34

age of onset:17

*

B. c.2019_2031del13 (exon 14)


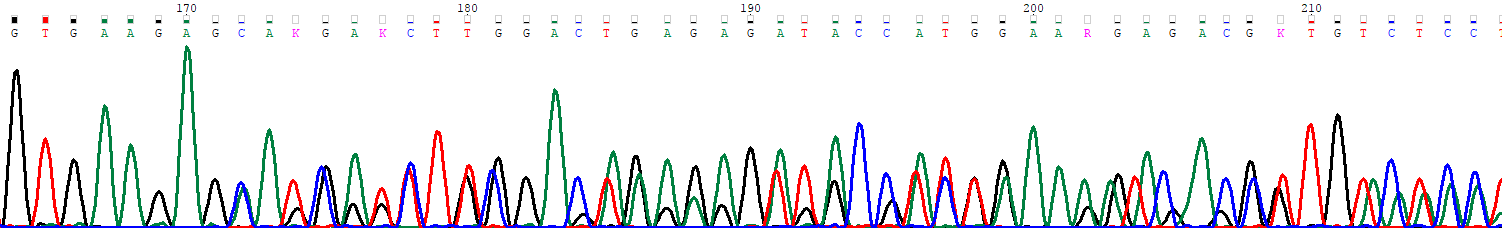


Supplementary Figure 9. Family ATH-53. A. Pedigree of patient ATH-53A (proband). Arrow indicates the proband. Affected and unaffected individuals are represented with black and open circles, respectively. Males are represented with a quadrant and females with a circle. The asterisk denotes the individual(s) genetically examined. B. Sequence chromatogram of the patient showing the novel frameshift mutation c.2019_2031del13 (p.Ser673Argfs*6) (Mut1) in heterozygosity. The arrow in B indicates the start of the frameshift caused by the deletion of 13 nucleotides (CATCGTCTTGGAG) marked by a horizontal line. This mutation was detected in compound heterozygosity with the previously reported missense mutation c.5882G>A (p.Gly1961Glu) (Mut2).

Supplementary Table 1: *ABCA4* PCR primer sequences.

| **Exon** | **Forward primer** | **Reverse primer** |
| --- | --- | --- |
| **1** | ATGCTTTGAAGGGGAAAAGTAGCC | ACAAGCCCACTGACTGCTCACAA |
| **2** | AGATACCACAACCAAAGTCCTACTGC | CATTGATAAATCTGTTACATGCATCA |
| **3** | AAATGCTGAGAATGAAGGAGGACATC | TAAGAGGTTAGGGGCTCAGCAAA |
| **4** | AGCTTGCAGTGAGCCGAGATCA | TGCCTCCGCTAGTATATTTTTCACC |
| **5** | AGAGACCCCAAGAACAGAACCTTCCT | ATATTTCTTGCCTTTCTCAGGCTGG |
| **6** | CCCATCTGCGATCTTAATTCCTGT | AAGGTCAATTCGTGAGGCTCTGCTA |
| **7** | GTAATCTTGCAGAAAAGATCCTGCG | TTGAAATTGCTAGATGGAAAGATCA |
| **8** | TGGATGTTTTCTTTTAAATGTGGAAG | AGGTTTGGTTTCACCTAGAAGTGTT |
| **9** | GAGTTGAATGAGACATGTGATGTGGA | ATGAGATGTGCTACCAGGAAGGCA |
| **10** | AAAAACACTTCCTTGAAAAGCCTTT | AAAGTCAGTGACCCCACTTGGTGA |
| **11** | AGCCTTAGCATCTCTATCCTCAATC | GTAGGGATTCTTGTTTCTTCAGTGGG |
| **12** | CTCTGGAGTTAAGCAAACATTAAGTTT | GTGACTCTGTAATTGCTTTCATTCAAGG |
| **13** | GAATTGGATCCTTTTGAAGCGT | TCTCCAATTTGGCTCTGGTCCCT |
| **14** | GAGGTTTTCCTGTTTTCCTTTCCCT | CACATGACTAATCCAGGCACATGAA |
| **15** | AGCACATGGAGTGTGCGTAGAAAT | GCCTCACGTGAACTTTTTAACCTTAG |
| **16** | TCCTGAGCTGACCTTACACTGAGA | GATTTAAACTTCAAATGCTGGTTCTG |
| **17** | AGCCTGAGAATAGCCATGTAATAATA | TGACTCATCAGGAATCACACCGTTT |
| **18** | GATTGTGTGATCAGGCTTGAGTACCT | TTGTCCACAGAGAGAGTCAGTTTCCT |
| **19** | ACGGTGATAGCCGAAGCCACAG | AGTAGACAGCCGCTGATAGGGAAA |
| **20** | TTTGAGTTTGACTGACAGCCCCA | CATAGGGGAAACCAAATAAGAGTCTG |
| **21** | TCCTCTTATTAGATATTTAAGCAGGGTC | AGTGCTCTGCAGGGAAAATGATCT |
| **22** | AACATCTAAGAGGCAGCACCAAAC | TCAGGAGGCTTTAGCTGGAACTTA |
| **23** | TCCTGATTTTTGCAACTATATAGCCA | CGAAATCTTCTGCAAATGGTCC |
| **24** | ATGTGTTGACTACACTTGGCAGTGAG | GAACAACTGTGTGACCTGCAGAAGTA |
| **25** | AAGTGGAGAGAAGTAGGAAATCTGGG | AATGTTAGACTTTTTCAAAGAACCG |
| **26** | GATGTTCTCAGATAGAGTCGTAATGG | ACTCAGGTGGTCCATCTGCCTT |
| **27** | CATTGCTGACTTAAATGAAGAGGAAA | TGAAGGAACACTCAGGAGAGGAGG |
| **28** | TGAAGGAACACTCAGGAGAGGAGG | TCATTGGTGAAGGTCCCAGTGAA |
| **29** | AGCATTCGGTGCCTCCAATACATA | AGAGACATGGAAGTGACCAGCAGA |
| **30** | TCTAAAGAGGAGGAGGAAAGGGTT | GCCAGTTTGAAATGTTAGTTTGTGAG |
| **31** | CAGCAGCTCAATCCATAAATAATTTT | TGCCCTGATCATACATAAATTGAGAG |
| **32** | CGGAAAATGCAAAAATGCCCCT | ATGGCTGTGAGGTGTGCCTTTTAA |
| **33-34** | CAATTCCCCCGAAAGTTCATGTT | GGATGGAATTTAATGAAGGTAGGAA |
| **35** | AAATCTAGCAAGGAATGTCTTCCAGC | TCGGATGTTCATATGTGCCTGACTA |
| **36** | GTTTAGTGGAGTGACAGCTTCAAGGT | ATACACAAGGCCCTTGGCCCAGT |
| **37** | AAGCAAGTCGAATCAGAATCCCAG | CGGAAGCTAAACTTGTGGGTGCTA |
| **38** | CAAATGGAGTATCTCTCTGGTCCCCT | AAGAAAGTGGCACTCATCCGCATA |
| **39** | AAATCCCTCCAGTGGCCAGTCT | TGCCACAGTCTGATGCAGGAGC |
| **40** | TTTGGCTCTTGCTCAGTTCCCA | TGTAGAAAAAACATTGTGGAGTGG |
| **41** | AAAGGATGGAAGCCCAGAAGGAA | GACCCTACATAAAACTGGGAACCAA |
| **42** | CTGCCCATGTCAATATGTAACCTCCT | GACTTGCATTATGGCATTATGTTCC |
| **43** | ATATGGCTCGTGGCCTCTGATG | TACAGATCTTTCAGGGCCTCAG |
| **44** | GCCAAATAGGAGAAGAGAAGAAGCAG | GGAATGAATGAATGAATAGCACGC |
| **45** | GAGCCAGCAGGAGCCTGTTTCA | TGTGAACCAAACACTGGGCTGAT |
| **46-47** | GAGTGACTGTGTGCGCCTTCTGT | CAGCAGGACTCTTCCAAGTGTCAAT |
| **48** | GCTGAGCTTAATCCCCAAAATTTC | TTATGCCTCCCTCTTATGGCAATTC |
| **49** | GTAGGACACAAGCCATACCAGCAG | TCTGTAGGAGGCATATCTGAGCCTT |
| **50** | GAGAGAAAGATGGCCCATAACCTG | TTTGTGATGAGTGCATTTGCATTTT |

Supplementary Table 2: Mutations detected in the *ABCA4* gene in a cohort of 59 Greek patients with presumed STGD1.

|  |  |  |  | **Allele 1** | | **Allele 2** | |  |
| --- | --- | --- | --- | --- | --- | --- | --- | --- |
|  | **ID N^o^ (Gender)** | **Age of onset** | **Exons**  **(Allele 1/Allele 2)** | **cDNA** | **Protein** | **cDNA** | **Protein** | **Μethod**  **(Allele 1/Allele 2)** |
| 1 | F24  (F) | 12 | IVS40/29 | c.5714+5G>A | - | c.4346G>A | p.Trp1449* | ABCR400 microarray |
| 2 | G21  (F) | 9 | IVS40/12,21 | c.5714+5G>A | − | c.1622T>C, c.3113C>T | p.Leu541Pro, p.Ala1038Val | ABCR400 microarray |
| 3 | H1  (F) | 40 | IVS40/IVS40 | c.5714+5G>A | − | c.5714+5G>A | − | ABCR400 microarray |
| 4 | H7  (M) | 22 | IVS40/4 | c.5714+5G>A | − | c.319C>T | p.Arg107* | ABCR400 microarray/Direct sequencing |
| 5 | ATH35  (F) | 35 | IVS40/IVS40 | c.5714+5G>A | − | c.5714+5G>A | − | ABCR400 microarray |
| 6 | ATH37  (F) | 5 | IVS40/12 | c.5714+5G>A | − | c.1622T>C | p.Leu541Prο | ABCR400 microarray |
| 7 | ATH79A  (F) | 20 | IVS40/12,21 | c.5714+5G>A | − | c.1622T>C, c.3113C>T | p.Leu541Pro, p.Ala1038Val | ABCR400 microarray |
| 8 | ABCA4-8A (F) | 15 | IVS40 | c.5714+5G>A | − | ND | ND | Direct sequencing/MLPA |
| 9 | ABCA4-12A (F) | 17 | IVS40/12,21 | c.5714+5G>A | − | c.1622T>C, c.3113C>T | p.Leu541Pro, p.Ala1038Val | Direct sequencing |
| 10 | ABCA4-15A (M | 23 | IVS40 | c.5714+5G>A | − | ND | ND | Direct sequencing/MLPA/ELOVL4 |
| 11 | ABCA4-27A (F) | 14 | IVS40/14 | c.5714+5G>A | − | c.2092T>C | p.Cys698Arg | Direct sequencing |
| 12 | ABCA4-29A (F) | NA | IVS40/4 | c.5714+5G>A | − | c.428C>T | p.Pro143Leu | Direct sequencing |
| 13 | C24  (M) | 30 | IVS40/12 | c.5714+5G>A | - | c.1622T>C | p.Leu541Pro | ABCR400 microarray/Direct sequencing |
| 14 | ABCA4-44A (M) | 40 | IVS40/IVS40 | c.5714+5G>A | − | c.5714+5G>A | - | Direct sequencing |
| 15 | ABCA4-47A (M | 40 | IVS40/3 | c.5714+5G>A | - | c.227A>C | p.Asn76Thr | Direct sequencing |
| 16 | ABCA4-53A (M) | NA | IVS40/13 | c.5714+5G>A | - | c.1819G>A | p.Gly607Arg | Direct sequencing |
| 17 | C22  (M) | 5 | 42/36 | c.5882G>A | p.Gly1961Glu | c.5087G>A | p.Ser1696Asn | ABCR400 microarray |
| 18 | D22  (M) | 30 | 42/30 | c.5882G>A | p.Gly1961Glu | c.4462T>C | p.Cys1488Arg | ABCR400 microarray |
| 19 | F8  (M) | 24 | 42/25 | c.5882G>A | p.Gly1961Glu | c.3812A>G | p.Glu1271Gly | ABCR400 microarray/Direct sequencing |
| 20 | F13  (M) | 16 | 42/8 | c.5882G>A | p.Gly1961Glu | c.868C>T | p.Arg290Trp | ABCR400 microarray |
| 21 | F25  (F) | 16 | 42/12 | c.5882G>A | p.Gly1961Glu | c.1622T>C | p.Leu541Pro | ABCR400 microarray |
| 22 | ATH57  (F) | 35 | 42/IVS29 | c.5882G>A | p.Gly1961Glu | c.4352+1G>A | - | ABCR400 microarray/Direct sequencing |
| 23 | ATH53  (M) | 17 | 42/14 | c.5882G>A | p.Gly1961Glu | c.2019_2031del13 | p.Ser673Argfs*6 | ABCR400 microarray/Direct sequencing |
| 24 | ATH76A  (M) | 6 | 42/12 | c.5882G>A | p.Gly1961Glu | c.1622T>C | p.Leu541Pro | ABCR400 microarray |
| 25 | ABCA4-1A (M) | 23 | 42/44 | c.5882G>A | p.Gly1961Glu | c.6112C>T | p.Arg2038Trp | Direct sequencing |
| 26 | ABCA4-2A (F) | 9.5 | 42/22 | c.5882G>A | p.Gly1961Glu | c.3322C>T | p.Αrg1108Cys | Direct sequencing |
| 27 | ABCA4-6A (F) | 22 | 42/12,21 | c.5882G>A | p.Gly1961Glu | c.1622T>C, c.3113C>T | p.Leu541Prο,  p.Ala1038Val | Direct sequencing |
| 28 | ABCA4-14A (M) | 14 | 42/IVS40 | c.5882G>A | p.Gly1961Glu | c.5714+1G>C | - | Direct sequencing |
| 29 | ABCA4-25A (F) | 16 | 42/22 | c.5882G>A | p.Gly1961Glu | c.3259G>A | p.Glu1087Lys | Direct sequencing |
| 30 | ABCA4-30A (F) | 17 | 42/23 | c.5882G>A | p.Gly1961Glu | c.3342delC | p.Met1115Cysfs*33 | Direct sequencing |
| 31 | ABCA4-32A (M) | 33 | 42/43 | c.5882G>A | p.Gly1961Glu | c.5917delG | p.Val1973* | Direct sequencing |
| 32 | ABCA4-46A (F) | 28 | 42/13 | c.5882G>A | p.Gly1961Glu | c.1819G>A | p.Gly607Arg | Direct sequencing |
| 33 | W16A  (M) |  | 42/28 | c.5882G>A | p.Gly1961Glu | c.4234C>T | p.Gln1412* | Direct sequencing |
| 34 | F11A  (M) | 13 | 12,21/12,21 | c.1622T>C, c.3113C>T | p.Leu541Pro, p.Ala1038Val | c.1622T>C, c.3113C>T | p.Leu541Pro, p.Ala1038Val | ABCR400 microarray |
| 35 | F11C  (F)  PATERNAL AUNT | 33 | 12,21/42 | c.1622T>C, c.3113C>T | p.Leu541Pro, p.Ala1038Val | c.5882G>A | p.Gly1961Glu | ABCR400 microarray |
| 36 | ATH44A  (M) | 6 | 12,21/IVS29 | c.1622T>C, c.3113C>T | p.Leu541Pro, p.Ala1038Val | c.4352+1G>A | − | ABCR400 microarray |
| 37 | ABCA4-34A (F) | 12 | 12/22 | c.1622T>C | p.Leu541Pro | c.3322C>T | p.Arg1108Cys | Direct sequencing |
| 38 | ABCA4-40A (F) | 12 | 12,21/6 | c. 1622T>C, c.3113 C>T | p.Leu541Pro, p.Ala1038Val | c.635C>T | p.Arg212Cys | Direct sequencing |
| 39 | ABCA4-52A (F) | 35 | 12,21/13 | c. 1622T>C, c.3113 C>T | p.Leu541Pro, p.Ala1038Val | c.1933G>A | p.Asp645Asn | Direct sequencing |
| 40 | E1  (M) | 20 | 9 | c.1140T>A | p.Asn380Lys | ND | ND | ABCR400 microarray/Direct sequencing/ MLPA |
| 41 | E5  (F) | 7 | 35 | c.4918C>T | p.Arg1640Trp | ND | ND | ABCR400 microarray/Direct sequencing/MPLA |
| 42 | H25  (M) | 6 | 43/43 | c.5917delG | p.Val1973* | c.5917delG | p.Val1973* | ABCR400 microarray |
| 43 | ATH73A  (F) | NA | IVS5/1 | c.571-2A>T | - | c.36G>A | p.W12* | ABCR400 microarray/Direct sequencing |
| 44 | ABCA4-5A (M) | 12 | IVS29/47 | c.4352+1G>A | - | c.6437G>A | p.Gly2146Asp | Direct sequencing |
| 45 | ABCA4-18A (F) | 8 | 6/22 | c.635C>T | p.Arg212Cys | c.3323G>T | p.Arg1108Leu | Direct sequencing |
| 46 | ABCA4-20A (F) | 13 | 1/35 | c.52C>T | p.Arg18Trp | c.4875T>A | p.His1625Gln | Direct sequencing |
| 47 | ABCA4-31A (F) | 7 | IVS40/IVS40 | c.5714+1G>C | − | c.5714+1G>C | − | Direct sequencing |
| 48 | ABCA4-35A (M) | 30 | 6/13 | c.658C>T | p.Arg220Cys | c.1819G>A | p.Gly607Arg | Direct sequencing |
| 49 | ABCA4-37A (F) | 15 | 1/IVS29 | c.52 C>T | p.Arg18Trp | c.4352+4A>C | - | Direct sequencing |
| 50 | ABCA4-39A (F) |  | 23/33 | c.3364G>A | p.Glu1122Lys | c.4771G>A | p.Gly1591Arg | Direct sequencing |
| 51 | W38A  (M) |  | 16/17 | c.2385_2400  delCTTACTGTCTCCGGTG | p.Ser795Argfs*43 | c.2626C>T | p.Gln876* | Direct sequencing |
| 52 | ABCA4-51A (F) | 8 | 13/44 | c.1819G>A | p.Gly607Arg | c.6077T>C | p.Leu2026Pro | Direct sequencing |

Supplementary Table 3: Polymorphic variants of the *ABCA4* gene detected in Greek STGD patients.

| **Number** | **Exon** | **Nucleotide change** | **Amino acid change** |
| --- | --- | --- | --- |
| 1 | 2 | c.141A>G  rs4847281 | p.Pro47Pro |
| 2 | Intron 3 | c.302+26A>G  rs2297634 | - |
| 3 | Intron 3 | c.302+101C>T  rs2297635 | - |
| 4 | Intron 3 | c.303-71delA  rs61753051 | - |
| 5 | Intron 3 | c.303-83dupT  rs569788505 | - |
| 6 | Intron 6 | c.769-86A>G  rs574741 | - |
| 7 | Intron 6 | c.769-32T>C  rs526016 | - |
| 8 | 6 | c.635G>A  rs6657239 | p.Arg212His |
| 9 | Intron 9 | c.1240-14C>T  rs4147830 | - |
| 10 | Intron 9 | c.1240-65delA  rs3215952 | - |
| 11 | Intron 9 | c.1239+71T>A  rs113931184 | - |
| 12 | 10 | c.1268Α>G  rs3112831 | p.His423Arg |
| 13 | 10 | c.1269C>T  rs4147831 | p.His423His |
| 14 | Intron 10 | c.1356+10dupG  rs4147887 | - |
| 15 | Intron 10 | c.1356+5_1356+6insG  rs281865386 | - |
| 16 | Intron 12 | c.1761-54G>A  rs4147833 | - |
| 17 | Intron 12 | c.1761-50G>A  rs61754022 | - |
| 18 | Intron 17 | c.2654-48G>C  rs12069723 | - |
| 19 | Intron 17 | c.2654-47T>C  rs12095320 | - |
| 20 | 19 | c.2828G>A  rs1801581 | p.Arg943Gln |
| 21 | Intron 27 | c.505-70T>A  rs143653650 | - |
| 22 | 28 | c.4203C>A  rs1801666 | p.Pro1401Pro |
| 23 | Intron 28 | c.4253+43G>A  rs61754045 |  |
| 24 | Intron 29 | c.4352+54A>G  rs547806 | - |
| 25 | Intron 32 | c.4668-58C>T  rs148896502 | - |
| 26 | Intron 33 | c.4773+48C>T  rs472908 | - |
| 27 | Intron 33 | c.4774-17_16delGT  rs55860151 | - |
| 28 | Intron 37 | c.5313-109A>G  rs56307710 | - |
| 29 | Intron 38 | c.5460+62G>A  rs2275033 | - |
| 30 | Intron 38 | c.5461-51delA  rs4147899 | - |
| 31 | Intron 38 | c.5461-51_5461-50delAG rs61754660 | - |
| 32 | Intron 38 | c.5461-49_5461-50insA rs61754663 | - |
| 33 | Intron 38 | c.5461-44dupG  rs281865391 | - |
| 34 | Intron 39 | c.5585-70C>T  rs537831 | - |
| 35 | 40 | c.5682G>C  rs1801574 | p.Leu1894Leu |
| 36 | 40 | c.5603A>T  rs 1801466 | p. Asn1868lle |
| 37 | 40 | c.5693G>A  rs1800552 | p.Arg1898His |
| 38 | Intron 40 | c.5715-25A>C  rs4147856 | - |
| 39 | 41 | c.5814A>G  rs4147857 | p.Leu1938Leu |
| 40 | Intron 41 | c.5836-43C>A  rs2275031 | *-* |
| 41 | Intron 41 | c.5836-11G>A  rs1800739 | *-* |
| 42 | 42 | c.5844A>G  rs2275029 | p.Pro1948Pro |
| 43 | Intron 43 | c.6006-16G>A  rs4147863 | *-* |
| 44 | Intron 43 | c.6006-85G>A  rs2275028 | *-* |
| 45 | Intron 43 | c.6006-81G>A  rs142316952 | *-* |
| 46 | 44 | c.6069T>C  rs1762114 | p.Ile2023Ile |
| 47 | Intron 45 | c.6282+7G>A  rs17110761 | - |
| 48 | 45 | c.6249C>T  rs1801359 | p.Ile2083Ile |
| 49 | 46 | c.6285T>C  rs1801555 | p.Asp2095Asp |
| 50 | Intron 48 | c.6729+21C>T  rs1800699 | - |
| 51 | Intron 48 | c.6729+51C>G  rs7518454 | - |
| 52 | Intron 48 | c.6730-3T>C  rs1800717 | - |
| 53 | 49 | c.6764G>T  rs6666652 | p.Ser2255Ile |
| 54 | Intron 49 | c.6816+28G>C  rs6666559 | - |
| 55 | 50  (3’ UTR) | c.*136G>A  rs55665437 | - |

| **Nucleotide change** | **Amino acid**  **change** | **Exon/Intron** | **PolyPhen-2** | **SIFT** | **PROVEAN** | **HSF** |
| --- | --- | --- | --- | --- | --- | --- |
| c.227A>C | p.Asn76Thr | 3 | Possibly Damaging (0.714) | Damaging  (0) | Deleterious  (3.391) | NA |
| c.2092T>C | p..Cys698Arg | 14 | Benign  (0.049) | Tolerated (0.14) | Deleterious (-2.676) | NA |
| c.4352+4A>C | NA | VS29 | NA | NA | NA | Alteration of the WT donor site, most probably affecting splicing |
| c.5714+1G>C | NA | IVS40 | NA | NA | NA | Alteration of the WT donor site, most probably affecting splicing |

Supplementary Table 4: Results of in silico analysis for 2 novel missense variants using the predictive algorithms SIFT, PolyPhen-2 and PROVEAN and for 2 novel splice variants using HSF prediction tool.
